# Learning curves for high tibial osteotomy using patient-specific instrumentation: a case control study

**DOI:** 10.1515/iss-2024-0007

**Published:** 2024-07-03

**Authors:** Davide Stimolo, Filippo Leggieri, Fabrizio Matassi, Angelo Barra, Roberto Civinini, Matteo Innocenti

**Affiliations:** Department of Orthopaedics, AOU Careggi Florence, 60222 University of Florence, Florence, Italy; Department of Technical Professions and Rehabilitation, AOU Careggi Florence, Florence, Italy; University of Florence, School of Human Health Sciences, Largo Brambilla 3, 50134 Florence, Italy

**Keywords:** high tibial osteotomy, HTO, PSI, personalized instrumentation, 3D planning, custom-made

## Abstract

**Objectives:**

Three-dimensional (3D) planning and Patient Specific Instrumentation (PSI) can help the surgeon to obtain more predictable results in Medial Opening Wedge High Tibial Osteotomy (mOW-HTO) than the conventional techniques. We compared the accuracy of the PSI and standard techniques and measured the learning curve for surgery time and number of fluoroscopic shots.

**Methods:**

We included the first 12 consecutive cases of mOW-HTO performed with 3D planning and PSI cutting guides and the first 12 non-supervised mOW-HTO performed with the standard technique. We recorded surgery time and fluoroscopic time. We calculated the variation (Δ delta) between the planned target and the postoperative result for Hip Knee Ankle Angle (HKA), mechanical medial Proximal Tibia Angle (MPTA), Joint Line Convergence Angle (JLCA) and tibial slope (TS) and compared it both groups. We also recorded the complication rate. We then calculated the learning curves for surgery time, number of fluoroscopic shots, Δ from target in both groups. CUSUM analysis charts for learning curves were applied between the two groups.

**Results:**

Mean surgical time and mean number of fluoroscopic shots were lower in PSI group (48.58±7.87 vs. 58.75±6.86 min; p=0.034 and 10.75±3.93 vs. 18.16±4.93 shots; p<0.001). The postoperative ΔHKA was 0.42±0.51° in PSI vs. 1.25±0.87° in conventional, p=0.005. ΔMPTA was 0.50±0.67° in PSI vs. 3.75±1.48° in conventional, p<0.001; ΔTS was 1.00±0.82° in PSI vs. 3.50±1.57° in conventional, p<0.001. ΔJLCA was 1.83±1.11° in PSI vs. 4±1.41° in conventional, p<0.001. The CUSUM analysis favoured PSI group regarding surgery time (p=0.034) and number of shots (p<0.001) with no learning curve effect for ΔHKA, ΔMPTA, ΔJLCA and ΔTS.

**Conclusions:**

PSI cutting guides and 3D planning for HTO are effective in reducing the learning curves for operation time and number of fluoroscopic shots. Accuracy of the procedure has been elevated since the first cases.

## Introduction

Osteotomies around the knee are effective joint-preserving surgical treatments for the painful osteoarthritis of the knee limited to one compartment and without bone-to-bone involvement. The most performed is medial open wedge high tibial osteotomy (HTO) for isolated medial arthritis determined by a proximal tibial extraarticular deformity. The aim is to shift the weight-bearing load on the lateral compartment relieving the medial one from overload. The surgeon’s final objective should be to postpone articular replacement as long as possible. The survival rate of the procedure after 10 years of follow-up is about 80–83 % [1, 2].

The surgeon must be extremely precise because inaccurate corrections can cause unbalanced load transfer, change of posterior tibial slope [3], and change of patella height [4, 5], thus results become unpredictable [6]. Free hand technique described by Lobenhoffer [7] is safe and effective, but it is technically demanding and requires a long learning curve. Literature reports the desired correction planned is not obtained in about ¼ of patients [8]. Although soft tissue laxity [9] and the cause of varus malalignment [10] can alter results, inaccurate execution of planned bone cuts can be one of the main causes of unsatisfying outcomes.

The desired correction on the coronal plane is planned on long-leg standing X-rays. It is accurate but it does not consider tibial slope and rotational changes [11]. Symmetrical or asymmetrical opening of the medial gap can change the tibial slope [3] but it is hard to precisely measure the sagittal correction in the intraoperative setting. Furthermore, no bidimensional imaging allows control over tibial rotation.

New technologies have been introduced to overcome the limits of the standard technique. Three-dimensional CT-based planning, intraoperative navigation systems [12] and patient specific instrumentation (PSI) were introduced. Recently, PSI technique associated with preoperative three-dimensional (3D) planning has been gaining favor. It consists of producing a patient specific cutting guide to be used during surgery to achieve the planned cut and gap opening. Several authors have introduced this technique in their practice with excellent results [13], [14], [15], [16]. In this article we describe our experience in medial opening wedge high tibial osteotomy (mOW-HTO) using PSI cutting guides.

We compared results in surgery time, number of fluoroscopic shots, correction obtained and learning curve between our first patients treated PSI assisted technique and patients treated with the standard free-hand technique. Our hypothesis was that PSI techniques could lead to accurate corrections and to lower the learning curves for surgery time and number of fluoroscopic shots.

## Materials and methods

This is a single center prospective case-control study. We included the first 12 consecutive cases of mOW-HTO performed with 3D planning and PSI cutting guides and the first 12 non-supervised mOW-HTO performed with the standard technique described by Lobenhoffer [7]. The patient’s attribution to one group was randomized. The power analysis calculation to detect significant results on learning curves determined that a minimum sample size of 20 participants for paired analysis would achieve 80 % power to detect a significant difference between groups. After an intermediate data analysis we demonstrated significant results after 12 cases. These results exceeded our expectations. Since we found the inflection point in the learning curve we decided to consider the results as conclusive. Indications for surgery are summarized in Table 1. All procedures were executed by the same young surgeon to reduce bias due to surgical technique and to measure the learning curves. All patients received long-leg standing X-rays in preparation for surgery. Patients undergoing PSI surgery received an additional CT scan according to the manufacturer’s technique (see below).

**Table 1:** Indications for medial opening wedge high tibial osteotomy.

Grade I–II–III K–L medial osteoarthritis
Varus knee <10°
Metaphyseal tibia vara MPTA <85°
Flexion contracture <10°
ROM 10–100°
Absence of persistent and significant patello-femoral pain
Stable in varus–valgus stress test

K-L, Kellgren-Lwarence Classification [17].

Inclusion criteria: consecutive cases, opening medial wedge HTO, single level osteotomies. Exclusion criteria: posttraumatic deformities, precedent surgery on the affected knee, associated anterior cruciate ligament reconstruction, severe rotational deformities, low quality post-operative long-leg standing X-rays, incomplete data. Age and BMI were no exclusion criteria.

We recorded surgery time and fluoroscopic time (number of fluoroscopic shots per surgery) in both conventional and PSI-assisted procedures. Between 6 weeks and 3 months from surgery, we performed long-leg standing X-rays. The desired correction angle was calculated using with the Miniaci [18] technique and was individualized for every patient. The aim was the weight-bearing axis passing through the Fujisawa point at 62.5 % of the tibial plateau [19].

To compare the correction targets we measured the following angles according to Paley [20, 21]: hip knee ankle angle (HKA), mechanical medial proximal tibial angle (MPTA), joint line convergence angle (JLCA), tibial slope (TS), on preoperative and postoperative long leg X-rays in both groups. JLCA was calculated as the angle between MPTA and m Lateral Distal Femoral Angle (LDFA). Tibial slope was calculated as the angle between the anatomic axis of the tibia and the joint line of the tibia in the sagittal plane [20, 21].

We calculated the variation (Δ delta) between the planned target and the result obtained regarding HKA, MPTA, JLCA and TS in both groups. We compared the Δ delta obtained between the PSI and the conventional groups. Postoperative X-rays were analyzed twice and independently by two of the authors. Final check of the measurements and the final opinion in case of discrepancy was carried out by the author (A.B.) who is radiology technician, responsible for CT scan procedures and dedicated to the orthopedics field. We also recorded the healing rate of the osteotomy and the complication rate in both groups. Patient consent was collected pre-operatively after they were informed of the procedure following the principles of the Declaration of Helsinki. The Ethics Committee of our Institution approved our study protocol before the investigation.

All statistical analyses were performed using SPSS software version 21 (SPSS Inc., Chicago, IL, USA). The Kolmogorov–Smirnov test was used to check the normal distribution of the continuous variables and thus the t-test was used for unpaired and paired continuous variables, and the chi-square test was applied for categorical variables. CUSUM analysis charts for learning curves were applied between the two groups regarding surgery time and the number of fluoroscopic shots needed to obtain the desired target of correction. Statistical significance was set at p <0.05.

### Conventional technique

In the conventional free hand technique described by Lobenhoffer [22] the surgeon’s objective is to reproduce the medial opening gap measured on preoperative templating [9, 18, 23]. Before surgery the surgeon should measure how much the gap should be opened in millimeters, bearing in mind that approximately 10 mm corresponds to 8–10 degrees of correction [24]. During the surgery the surgeon has at least two methods to verify the amount of correction: 1 – measuring the gap opening with a ruler; 2 – fluoroscopic control of the lower limb axis, with the new axis passing through the lateral inclination of the lateral tibial spine. Tibial slope can be modified according to preoperative planning [25]. The surgeon needs to get symmetrical opening in the anterior and posterior part of the gap if the aim is to maintain the slope unaltered, the aim is to get asymmetrical gap opening in the anterior and posterior side of the osteotomy if the aim is to correct the native slope [26, 27].

### PSI cutting-guides technique

All patients listed for PSI mOW-HTO receive an additional preoperative CT scan to the conventional long-leg standing X-rays. CT scans are executed with the Activemotion CT protocol that involves a bilateral acquisition (right and left leg) of fields of view with the same spatial coordinates: 2 cm long package (with 2 mm slices) located on the heads of the femurs, 15 cm long package (with 0.6 mm slices) from distal femur to proximal tibia; 5 cm long (with 0.6 mm slices) on the ankles with soft tissue viewing window. After bidimensional templating the surgeon sends the company the description of the correction desired on coronal and sagittal planes. The company sends back a 3D reconstruction of the lower limb reproducing native alignment and new coronal and sagittal alignment after the planned HTO. The surgeon can then decide if further modifications are required or not.

**Figure 1:**
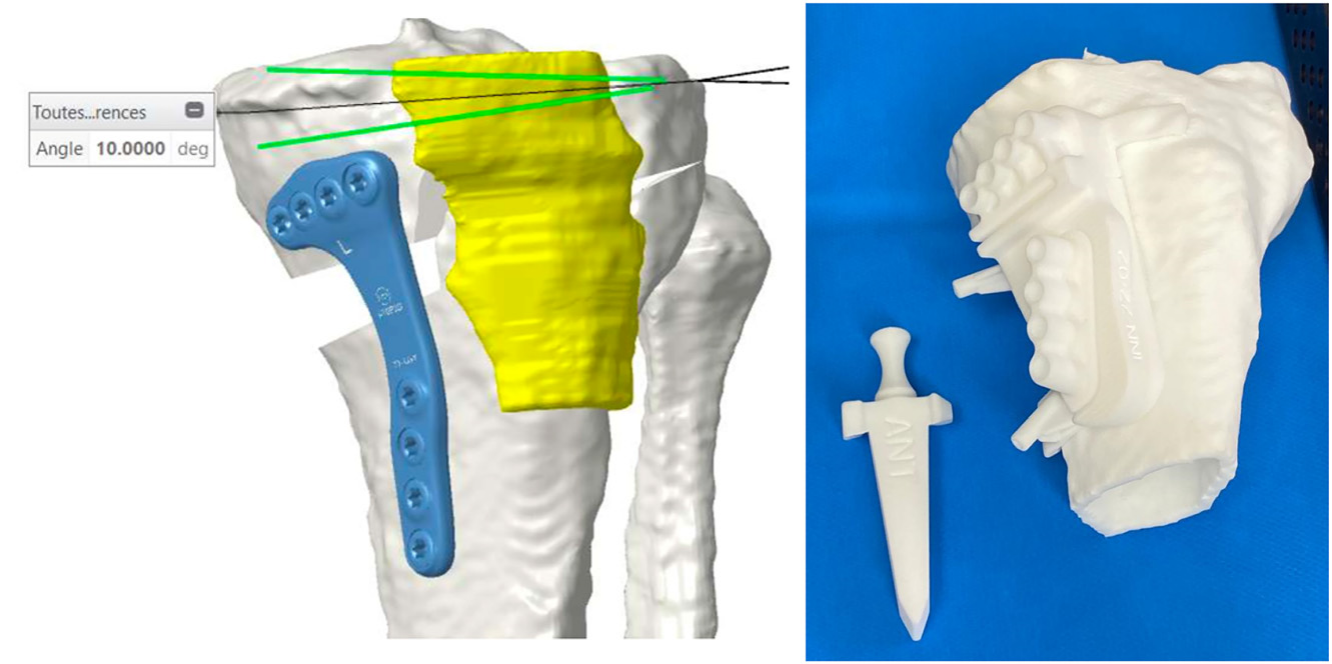
(a) Preoperative 3D planning of correction and plate positioning; (b) preoperative template of patient specific instrumentation: proximal tibia of the patient, personalized cutting guide with holes to drill for plate positioning, opening wedge.

After that the manufacturer produces the PSI instrumentation. (Newclip Technics, Haute Goulaine, France). It includes personalized cutting jigs, 3D bone model, patient-specific wedge to open the osteotomy cut. Cutting guides are made of biocompatible plastic material Nylon-PA2200. All the PSI are sterile and available for intraoperative use (Figure 1a and b). The company takes 5 weeks to produce the cutting guides from the moment the CT is performed. The cost of the procedure in our hospital is around EUR 1500 including CT scan, PSI instruments, cutting guides, and fixation devices (plate and screws).

**Figure 2:**
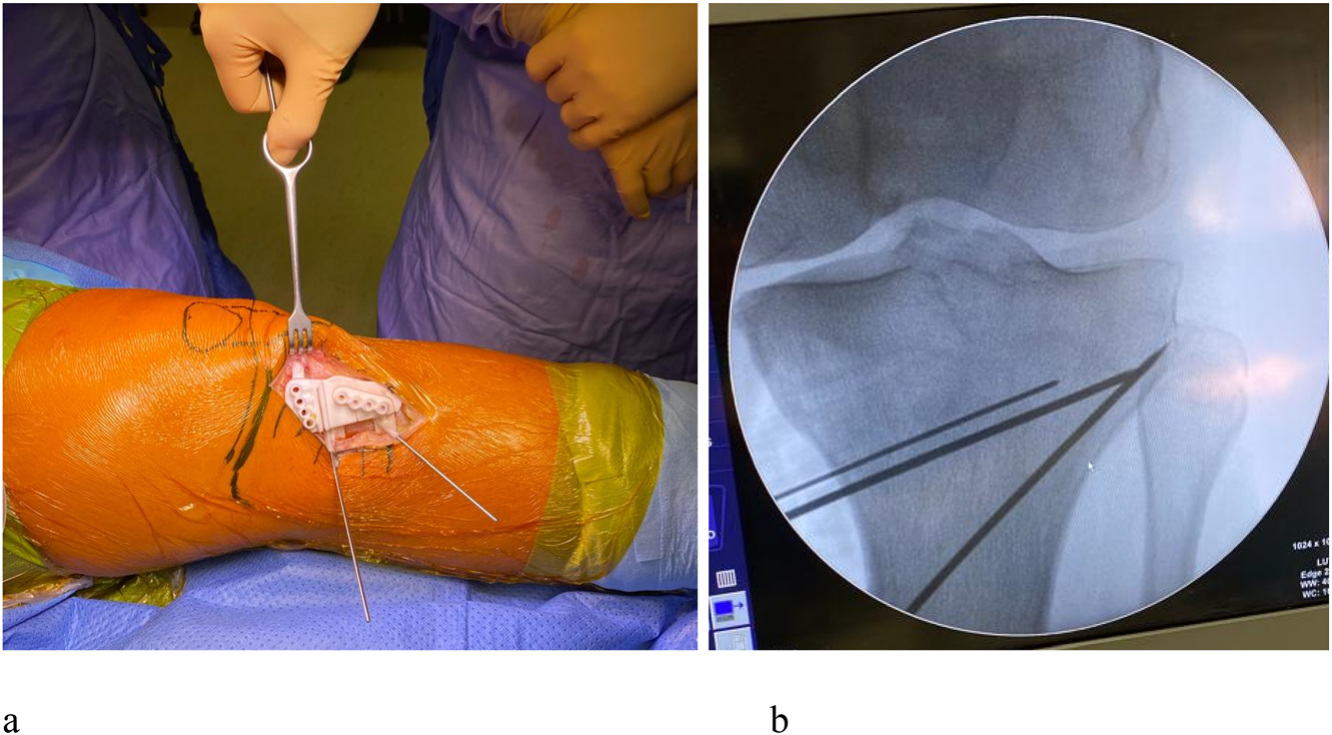
(a) Proximal tibia exposure, personalized cutting guide positioned and stabilized with K-wires; (b) fluoroscopic control of guide position and appropriate depth of K-wire introduction to avoid hinge fractures.

**Figure 3:**
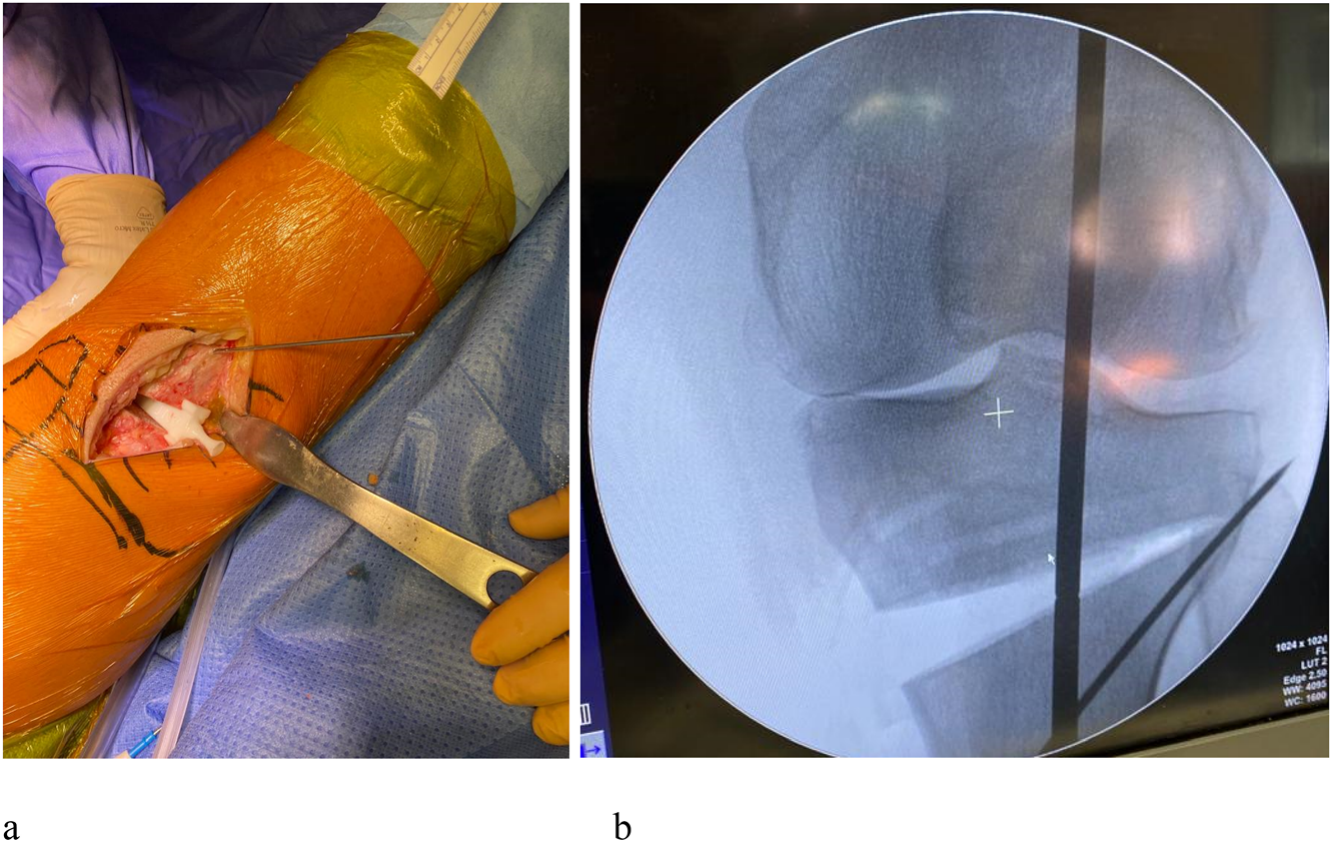
(a) Opening of the medial wedge; (b) fluoroscopic control of the alignment obtained. The use of a custom-made 3D wedge helps to achieve an accurate opening as planned.

**Figure 4:**
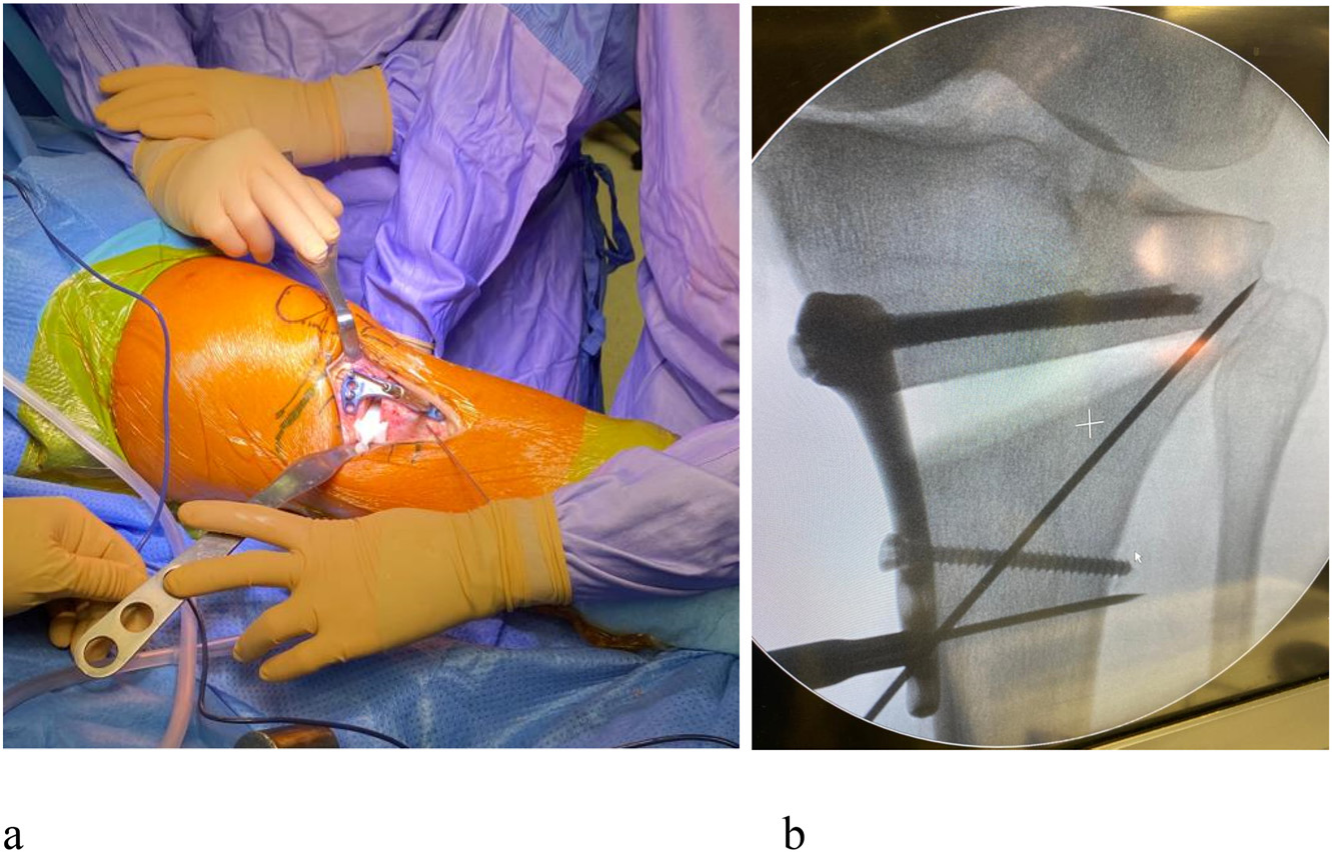
(a) Plate positioning; (b) fluoroscopic control of correct plate positioning.

In the operatory room, the axis of the lower limb is controlled under fluoroscopy before the operation. A standard medial exposure to the proximal tibia is performed, and the tibial bony surface is exposed. The cutting jig is positioned on the bone and the guided K-wires are introduced (Figure 2a and b). If fluoroscopy confirms the correct position of the cutting guide the holes for the plate are drilled and the osteotomy is performed. Afterward, the medial gap is gradually opened (Figure 3a and b), and the osteotomy is fixed with a locking plate (Newclip Techniques, Haute Goulaine, France) (Figure 4a and b). The final lower limb alignment and the plate position are checked under fluoroscopic control before closure.

The postoperative rehabilitation protocol included immediate active motion in a hinged brace and toe-touch weight bearing for 2 weeks for pain control. Afterward, progressive weight bearing as tolerated was allowed.

## Results

We collected the 12 cases of PSI HTO and 12 cases of conventional techniques HTO in the time frame 2021–2022. The populations were comparable based on male:female ratio, BMI mean HKA, mean MPTA (Table 2).

**Table 2:** Patient population PSI and traditional technique (TT) groups.

PSI	SEX	BMI	HKA	MPTA	TT	SEX	BMI	HKA	MPTA	
1	M	24	171	81	1	F	22	174	83	
2	M	22	173	84	2	F	26	172	82	
3	F	26	173	83	3	M	24	170	82	
4	M	27	172	83	4	M	23	168	80	
5	F	23	174	85	5	M	22	169	81	
6	M	21	170	83	6	F	25	172	83	
7	F	24	171	82	7	M	24	173	83	
8	M	25	174	84	8	M	27	174	84	
9	M	22	169	81	9	M	25	172	83	
10	M	26	172	84	10	F	23	170	82	
11	M	24	171	83	11	F	24	171	81	
12	M	21	171	80	12	M	22	173	84	
**SEX**		**M9:F3**				**M7:F5**				**p=0.38**
**BMI**		**23.75±2.00**				**23.91±1.62**				**p=0.82**
**HKA**		**171.75±1.54**				**171.5±1.93**				**p=0.72**
**MPTA**		**82.75±1.48**				**82.3±1.23**				**p=0.46**

At the bottom-lines M:F ratio and mean values with standard deviation for BMI, HKA, and MPTA. Difference between the two populations was not statistically significant (significant p-value: p<0.05).

The mean surgical time was 48.58±7.87 min in PSI and 58.75±6.86 min in conventional technique patients, p=0.034. The mean number of fluoroscopy shots was 10.75±3.93 (range 6–18) in PSI group and 18.16±4.93 (range 12–28) in conventional technique, p <0.001.

The postoperative mean HKA was 181.75±1.14° in the PSI group and 180.5±1.57° in the conventional, Δ from planned 0.42±0.51° in PSI vs. 1.25±0.87° in conventional, p=0.005.

The mean postoperative MPTA was 92.5±1.1° in PSI group and 89.5±1.8° in the conventional group, Δ from planned 0.5±0.67° in PSI vs. 3.75±1.48° in conventional, p<0.001; postoperative mean TS was 9±1.8° in PSI and 10±1.4° in conventional, Δ from planned 1.00±0.82° in PSI vs. 3.50±1.57° in conventional, p <0.001 (Table 3). Regarding the JL the difference from planned was 1.83±1.11° in PSI vs. 4±1.41° in conventional, p <0.001.

**Table 3:** Mean values of correction for HKA, MPTA and TS and delta from planning in PSI and conventional groups.

	HKA			MPTA			TS		
	PSI	CONV	p	PSI	CONV	p	PSI	CONV	p
Post-op	181.75±1.14°	180.5±1.57°		92.5±1.1°	89.5±1.8°		9±1.8°	10±1.4°	
Δ from planning	0.42±0.51°	1.25±0.87°	0.005	0.5±0.67°	3.75±1.48°	p<0.001	1.00±0.82°	3.50±1.57°	p<0.001

T-student analysis of delta obtained between the two groups. p values for difference in delta values.

In both group we report complete healing of the osteotomy in 100 % of cases and no cases of hinge fractures. In the PSI group we recorded two postoperative hematomas not requiring surgical evacuation, one superficial tissue infection treated with antibiotics and advanced local medications, 2 cases of local pain and discomfort which required hardware removal after complete healing of the osteotomy. In the conventional group we recorded 1 case of local hematoma, zero soft tissue infections and 2 cases of local pain requiring hardware removal. Chi square test was not statistically significant at p=0.38.

**Figure 5:**
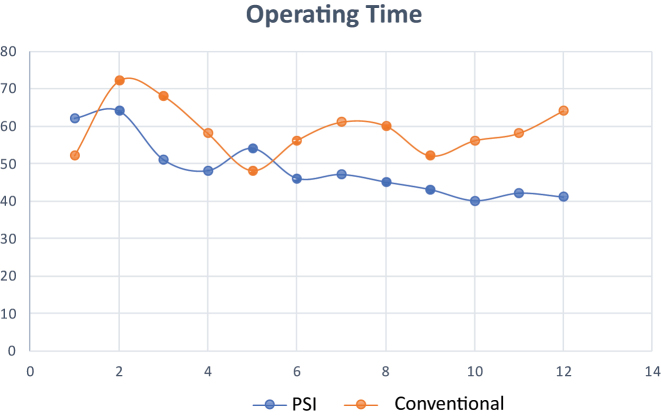
CUSUM analysis charts for learning curves for operating time: a sharp inflexion was observed after 6 cases separating the learning phase to the proficiency phase for the PSI group no inflection and persistency was found for the conventional group during the first 12 procedure. p=0.0034.

**Figure 6:**
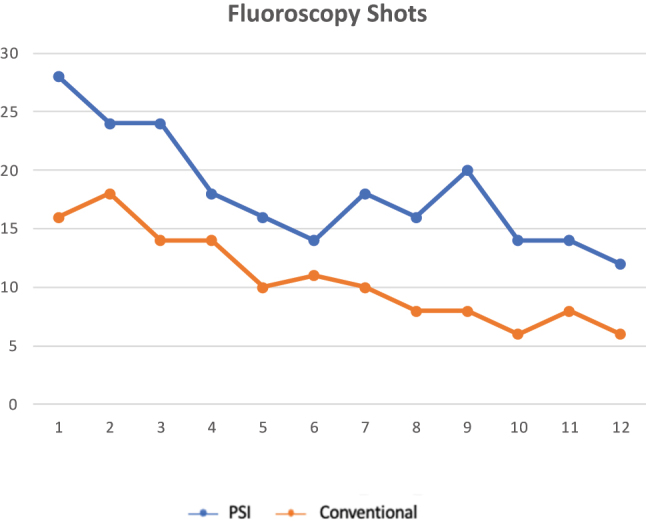
CUSUM analysis charts for learning curves for number of fluoroscopy shots: 5 cases for PSI group and 10 cases for conventional group separated the learning phase to the proficiency phase for the PSI group. p<0.001.

The CUSUM analysis charts for learning curves regarding surgery time demonstrated a sharp inflection after 6 cases separating the learning phase to the proficiency phase for the PSI group but no inflection and persistency were found for the conventional group (Figure 5). Regarding the number of fluoroscopy shots, it took 5 cases for PSI to reduce the number of shots and 10 cases for the conventional group (Figure 6).

**Figure 7:**
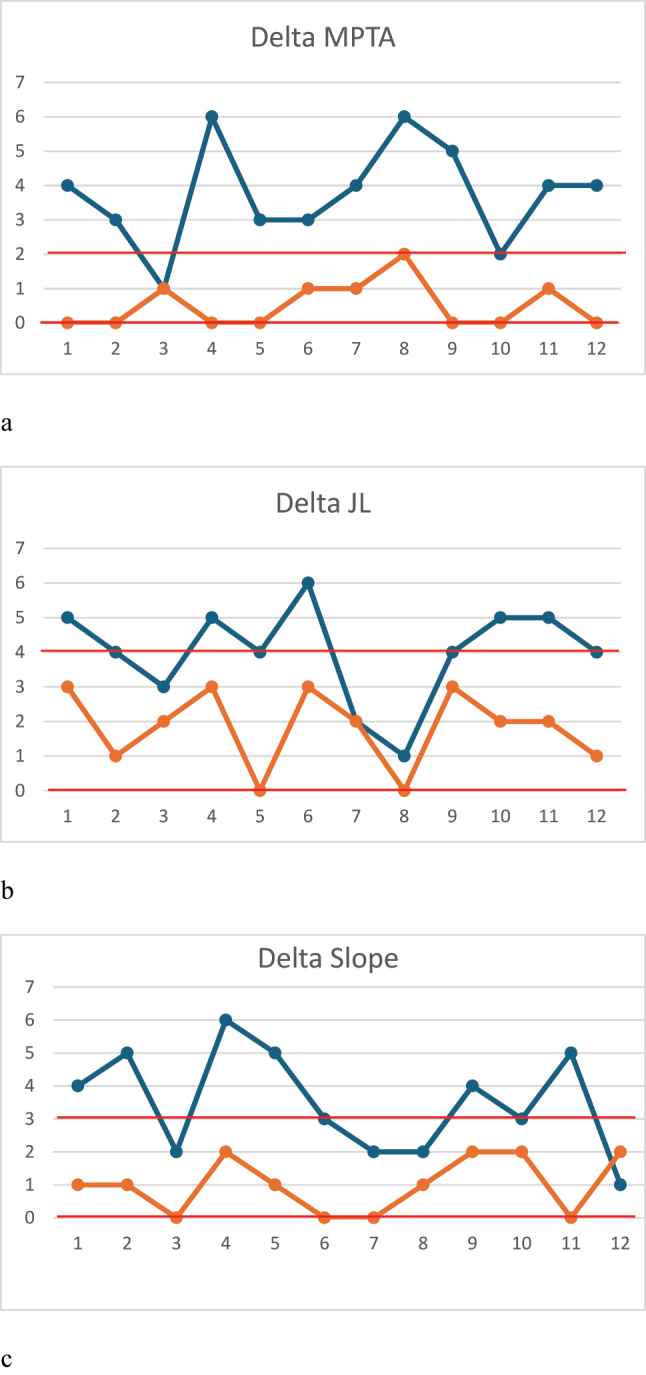
CUSUM analysis charts for learning curves: (a) delta MPTA p<0.001; (b) delta JL p<0.001; (c) delta TS p=0.00031.

CUSUM analysis charts for learning curves for ΔHKA, ΔMPTA, ΔJL and ΔTS demonstrated no learning curve effect in the PSI group. It was not possible to define any learning curve in the conventional group (Figure 7a–c).

## Discussion

The most evident results of our study are related to surgery duration and the number of fluoroscopic shots needed to reach the correction targets. The surgery time was shorter and the number of shots was lower for the PSI cutting-guides group compared to the conventional group. In the literature other studies support the quick learning curve and reduced operating time for PSI [15, 28]. Jacquet et al. [29] showed the learning curve of 10 cases to reduce operating time, 9 cases to reduce fluoroscopy shots, and 8 cases to surgeons’ anxiety levels before the procedures. In addition, they demonstrate that the capability to get the desired correction target is not affected by the learning curve, and accurate results are achieved from the first cases. Our study is lower in numbers, but it is a case-control study and its strength is the comparison between patients who have undergone PSI-assisted surgery vs. conventional surgery. The CUSUM analysis showed 6 cases to step to the proficiency level for the PSI technique related to the operating time and no inflection of the curve after 12 procedures for the conventional techniques. The CUSUM analysis for fluoroscopy use demonstrates the necessity of 10 cases to reduce the use of fluoroscopy in the standard technique vs. five cases in the PSI group.

There is concern about the safety of the procedure. Studies including a large number mOWHTO practiced with the traditional free hand technique demonstrate an unexpected number of cases in which the desired target of correction is missed even in experienced hands [30]. Van den Bempt et al. in a systematic review including 966 patients revealed that in eight out of 14 studies the accepted range of correction was under 75 % [8]. In our study we noticed a tendency to under-correction in the conventional technique group. On the contrary, we demonstrate accurate correction in PSI group regarding HKA, MPTA, JLCA and TS with postoperative targets obtained very close to the aims. Many studies in the literature support the necessity of patient-specific instrumentation to get more accurate results. In a cadaveric study, Donnez [31] obtained a difference from the planned of 0.2° (max 0.5°, SD 0.3°) in the frontal plane and − 0.1° (max 0.8°, SD 0.5°) in the sagittal plane by using patient-specific cutting guides. Victor et al. [14] demonstrated an accuracy on HKA of 0.3±0.75°. A large observational study [13] including 100 patients demonstrated on postoperative CT scan no significant differences between planned and obtained HKA and MPTA using PSI (mean ΔHKA was 1±0.95°, the mean ΔMPTA was 0.54±0.63°). A recent RCT compared desired vs. correction for HKA in patients randomized among three groups: conventional technique, intraoperative navigation technique and PSI. They showed more accurate correction of HKA in the PSI group (p=0.001) [32].

Other studies, on the contrary, do not demonstrate the superiority of the use of PSI. In a systematic review [33] the authors demonstrate more outliers in the conventional treated patients than in the PSI patients (40 vs. 15 %) but not a statistically significant higher accuracy (p=0.98). Another study [34] does not demonstrate higher accuracy in the accuracy to obtain the desired HKA (p=0.41) and MPTA (p=0.64) between PSI and standard technique. However unexpected outcomes do not only depend on the accuracy of bone cuts. Soft tissue laxity of the lateral collateral ligament should be considered in planning [9] otherwise it can lead to over or under-correction. CT scan is taken in supine position and it does not take into account the load bearing effect on lower limbs alignment, so that the 3D planning could not be completely predictable of final results. Nejima et al. demonstrated how templating on supine X-rays could underestimate the correction desired [35].

Discussing the safety of the procedure, in literature it is not possible to say the traditional technique is safer than PSI [36]. In our series healing rate, soft tissue complications, hardware removal were comparable between the two groups. A systematic review [37] including 71 studies and 7,836 patients report 9.1 % of lateral hinge fractures, 2.2 % of soft tissue infections, 1.1 % of nonunions and 10–15 % of hardware removal. Our series is limited in number and has a limited follow up but we can report lower intraoperative fractures, nonunions, and soft tissue infection (0.08 vs. 2.2 %); however our reoperation rate due to implant removal was 20 % in both groups.

The concerns about PSI are mainly due to a couple of reasons: radiation exposure and costs. Radiation exposures are related to the preoperative CT scan. ActiveMotion is a CT scan protocol which is limited to anatomical segments of interest, but the exposure is DLP 1799 mGy/cm (Dose Length Product). We try to compensate by lower number of fluoroscopic shots in the operating room but the exposure is still higher with the CT scan. The advantage could be in patients who necessitate bilateral procedures because we can make only one CT scan and template both the osteotomies. Costs are a big issue. In our hospital, an osteotomy performed with PSI technique costs about 1,500 euros (including CT scan). The traditional technique costs about 300 euros per procedure. The cost of one PSI covers about five traditional procedures. However, we need to consider that lower operation time means lower costs. In our hospital the operatory room costs about 40 euro/min, we measured a difference of about 10 min in favor of PSI so about 400 euros of reduced indirect costs. Considering this, one PSI covers about 2–3 conventional HTO instead of 5. If the two techniques had comparable costs, we believe it would be worth using a technique that is safe, faster, accurate and reduces radiation exposure for the patient and surgical equipe. Moreover, if long-term studies will confirm that the more accurate results can delay or avoid the implant of TKA it would be a great saving on money for each patient and higher functional results. Up to now, in the literature, HTO for patients under 60 years old is considered more cost-saving operation than unicompartimental or total knee arthroplasty [38]. Currently in our institution with a view to optimising economic resources, PSI technique is currently used by junior specialists to gain experience in osteotomies safely without reducing accuracy of results. Experienced surgeons used the technique to learn it, now they only use it for complex or challenging cases like double level osteotomies or post-traumatic deformities.

## Limitations

Our study is a single center, single surgeon small series. Its strength is the case-control design but its limitation is the low number of patients included. Moreover, postoperative evaluation of HKA, MPTA, TS and JLCA is measured on X-rays, which is less precise than a postoperative CT scan. In the postoperative bidimensional analysis we cannot describe effect on tibial rotation that can be responsible for less predictable results [11]. We picked a young surgeon at his first experience out of training in the field of osteotomy around the knee, and this can rise surgery time and number of fluoroscopic shots needed. However, we must consider that comparing the learning curve for a new surgical technique with surgeons already experienced with the conventional one could be misleading. The risk is to underestimate the difference in learning curves. That’s why, we compared the first 12 cases with the PSI technique and the first non-supervised 12 cases with the conventional technique performed during the same time frame. Therefore, in our study, the surgeon had similar numbers and experience in HTO surgery performed with the conventional and the PSI techniques so the learning curves for the two procedures are comparable.

## Conclusions

PSI cutting guides and 3D planning for HTO are effective in reducing the learning curves for operation time and the number of intraoperative fluoroscopic shots. The accuracy of the procedure has been elevated since the first cases. The complication rate is low and comparable to the conventional technique. We need long-term data to demonstrate a lower failure rate and conversion to total knee arthroplasty and so to justify higher radiation exposure and costs.
